# Basic life support is effectively taught in groups of three, five and eight medical students: a prospective, randomized study

**DOI:** 10.1186/1472-6920-14-185

**Published:** 2014-09-06

**Authors:** Moritz Mahling, Alexander Münch, Sebastian Schenk, Stephan Volkert, Andreas Rein, Uwe Teichner, Pascal Piontek, Leopold Haffner, Daniel Heine, Andreas Manger, Jörg Reutershan, Peter Rosenberger, Anne Herrmann-Werner, Stephan Zipfel, Nora Celebi

**Affiliations:** Medical School, Faculty of Medicine, University of Tübingen, Geissweg 5, Tübingen, 72076 Germany; Department of Anesthesiology and Intensive Care Medicine, University of Tübingen, Hoppe-Seyler-Straße 3, Tübingen, 72076 Germany; Department of Internal Medicine VI, Psychosomatic Medicine, University Hospital of Tübingen, Osianderstraße 5, Tübingen, 72076 Germany; Ärztezentrum Ostend, Ostendstr. 90, Stuttgart, 70188 Germany

## Abstract

**Background:**

Resuscitation is a life-saving measure usually instructed in simulation sessions. Small-group teaching is effective. However, feasible group sizes for resuscitation classes are unknown. We investigated the impact of different group sizes on the outcome of resuscitation training.

**Methods:**

Medical students (n = 74) were randomized to courses with three, five or eight participants per tutor. The course duration was adjusted according to the group size, so that there was a time slot of 6 minutes hands-on time for every student. All participants performed an objective structured clinical examination before and after training. The teaching sessions were videotaped and resuscitation quality was scored using a checklist while we measured the chest compression parameters with a manikin. In addition, we recorded hands-on-time, questions to the tutor and unrelated conversation.

**Results:**

Results are displayed as median (IQR). Checklist pass rates and scores were comparable between the groups of three, five and eight students per tutor in the post-test (93%, 100% and 100%). Groups of eight students asked fewer questions (0.5 (0.0 – 1.0) vs. 3.0 (2.0 – 4.0), p < .001), had less hands-on time (2:16 min (1:15 – 4:55 min) vs. 4:07 min (2:54 – 5:52 min), p = .02), conducted more unrelated conversations (17.0 ± 5.1 and 2.9 ± 1.7, p < 0.001) and had lower self-assessments than groups of three students per tutor (7.0 (6.1 – 9.0) and 8.2 (7.2 – 9.0), p = .03).

**Conclusions:**

Resuscitation checklist scores and pass rates after training were comparable in groups of three, five or eight medical students, although smaller groups had advantages in teaching interventions and hands-on time. Our results suggest that teaching BLS skills is effective in groups up to eight medical students, but smaller groups yielded more intense teaching conditions, which might be crucial for more complex skills or less advanced students.

**Electronic supplementary material:**

The online version of this article (doi:10.1186/1472-6920-14-185) contains supplementary material, which is available to authorized users.

## Background

Cardiac arrest remains the leading cause of death in Europe [1]. Without treatment, the cardiac arrest survival rate declines by 5.5% per minute [2]. Early and effective cardiopulmonary resuscitation (CPR) can improve patient prognosis [1]. However, in reality CPR quality is reported to be poor even for health-care professionals [3]. Therefore, it is essential that medical students learn CPR as part of their curriculum. Simulation training is able to improve student skills and might improve patient outcomes [4, 5].

The time tutors can spend with students and the availability of resuscitation simulators is limited. Therefore, understanding the impact of assigning a defined number of students to a tutor and simulator is vital. Problems associated with larger groups might be that the “teacher gives a lecture rather than conducting a dialog” and that “students cannot be encouraged to talk except with difficulty” [6]. Tutors may believe that students in smaller groups perform better and therefore perform better themselves (*Pygmalion effect*) [7]. Smaller groups could benefit from a more intensive dialog with their tutor, which could lead to more interaction in terms of questions and tutor interventions. Most studies investigating the influence of group size on student satisfaction show a preference for small group teaching [8–12]. On the other hand, students in larger groups can observe other classmates performing resuscitation. Observational learning is a major contribution to the acquisition of motor skills and could therefore provide an advantage for students in larger groups [13].

At this time, the effect of different sizes of groups on the effectiveness of CPR simulation training remains unclear. Rezmer *et al.* recently investigated the impact of different group size on a post-simulation oral exam and questionnaire [14]. However, they only investigated relatively small and comparable groups of two to four students and did not use outcomes related to the resuscitation quality or teaching session itself. Although studies using problem-based education found that there was less student participation in larger groups, there remains a lack of knowledge considering medical education [15].

This knowledge gap is surprising as specifying the optimal group size is an everyday task in paramedic education, in public training and at medical schools. Therefore, we investigated the influence of different sizes of group including three, five or eight students per tutor on success of basic life support (BLS) teaching, important resuscitation quality features and the teaching session itself. We furthermore assessed self-perception of the participants in order to investigate the discrepancy between objective and subjective measures.

## Methods

### Study design and setting

We performed a prospective, randomized and double-blind simulation study. This study was conducted in the 2012/2013 winter term at the Medical Faculty of the University of Tübingen, Germany. The term “simulation” refers to the general simulation of a situation using a manikin, while the contents of the BLS training were defined according to the 2010 ERC guidelines [1].

### Participants and randomization

**Figure 1:**
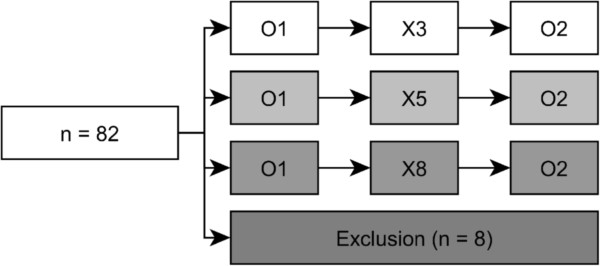
**Randomization flow-chart.** Flow-chart of all 82 participants entering the randomization. Between the first and second observation (O1 and O2), students received the BLS training intervention in the assigned group size (X3, X5, X8).

A total of 123 fourth year medical students participating in a resuscitation class were asked to take part in this study. Thirty-six students declined and five students were excluded because of health complaints. Eighty-two students were included in the randomization process using the sealed envelope system. Eight students were randomly excluded to standardize group sizes. A total of 74 medical students were allocated to groups of three (X3, n = 30), five (X5, n = 20) or eight (X8, n = 24) students per tutor (Figure 1).

### Basic life support training

Each group of students participated in BLS training provided by one of four trained tutors. The tutors were experienced in teaching emergency medicine and followed a standardized curriculum. Two tutors were medical students with paramedic experience, and two were anesthesia nurses. All tutors were blind to the intervention and outcome parameters. The tutors were permuted among the different group sizes to avoid a tutor bias. Each tutor was assigned to one group and taught his three, five or eight students in a separate room.

The training comprised a theoretical introduction for five minutes followed by a practical demonstration of assessment, chest compression and bag-mask ventilation each for five minutes. The tutor was provided a detailed instruction about the structure and contents of the training in order to standardize teaching (Additional file 1). Throughout the training, he guided the participants, provided feedback and answered questions. Each student was allowed to train assessment, chest compressions and bag-mask ventilation individually before practicing a comprehensive BLS resuscitation. The total training time was calculated to facilitate the amount of participating students (20 minutes introduction + 6 minutes practical training per participant, including hands-on training as well as time needed for rotation, familiarization and individual questions) and was videotaped and audiotaped from two different angles (Samsung Full-HD-Camcorder, Samsung Electronics GmbH, Schwalbach, Germany).

### Objective structured clinical examination measurements

Before (pre-test) and after (post-test) receiving the BLS training detailed above, all participants had to conduct an objective structured clinical examination (OSCE) to assess their BLS skills [16]. The BLS training and the OSCE took place on the same afternoon. The students were asked to perform BLS resuscitation on a manikin. The instruction included information about the patient and about the required task, but no details about how these skills should be performed. Instructions were read to the participants, and an additional printed instruction sheet was available for the participants. OSCE sessions were videotaped and audiotaped from two different angles, and resuscitation performance was measured using a Resusci Anne Advanced Skill Trainer (Laerdal Medical GmbH, Puchheim, Germany).

### Outcomes

The primary outcome was performance measured by a modified *Brennan* checklist that was updated to the European Resuscitation Council 2010 guidelines (ERC 2010) [17, 18] (Additional file 2). The checklist consisted of 14 equally weighted items [19]. All items were rated on a four-point scale where four points is indicative of the best score and one point is the lowest achievable score. The checklist included the assessment of the general approach, assessment, chest compression and ventilation. The quality of chest compression was calculated using objective manikin data. To pass the OSCE, a participant had to earn at least three points on average for the checklist (in total 42 out of 56 points). The checklist was validated by two physicians and is available as online supplemental material. Two trained paramedics blinded to the study question and intervention rated the participants using the video and audio recordings. If there was significant internal disagreement, videos were discussed until a consensus was found.

A secondary outcome was the objective resuscitation quality as proposed by Kramer-Johansen *et al.* [20]. We measured the mean compression rate, compression depth, “duty cycle” and the percentage of compressions completely released using the Laerdal PC Skill Reporting System (Version 2.4.1, Laerdal Medical GmbH). We furthermore report the percent of students whose mean chest compression depth and rate was within the ERC recommended ranges. Breathing parameters were not included due to technical problems with the manikin.

During the BLS training session we recorded the number of questions asked by each participant, the amount of tutor interventions (e.g. hand placement corrections) per participant and the effective hands-on time of each individual participant defined by the time actively spent with the manikin. Question/answer dialogues were defined as “a dialog between tutor and trainee. It consists of a question from the trainee to the tutor and its matching answer by the tutor. Every question is counted”. Comments not related to the resuscitation training (“unrelated conversations”) were counted for each group of students. These variables were also reported by the two video raters.

Although the students received identical teaching regardless of the group size, we also collected data on their self-assessment in order to measure the Hawthorne-effect. Before and after the training, all participants were asked to assess their BLS skills on a continuous numeric scale from zero to ten with ten being the best self-assessment. In addition, we asked for potential confounders during the pre-assessment (age, gender, previous training as a paramedic or other training, month elapsed since the last resuscitation training).

### Statistical analysis

Variables following a Gaussian (normal) distribution are expressed as mean and standard deviation (SD). Non-normal distributed variables are indicated as median and interquartile ranges (IQR). The Kruskal–Wallis analysis was used to test multiple groups, the Wilcoxon signed-rank test to compare two paired samples and the Mann–Whitney *U* test to compare two unpaired values. In all other cases two-tailored Student t-tests were used.

We calculated the interclass correlation (ICC) for each human-rated checklist item to assess the inter-rater agreement, based on the results of each individual student [21]. As we measured individual skills, all statistical calculations are related to individual participants if not stated otherwise. A p-value < .05 was considered statistically significant. If multiple groups were compared, we used Bonferroni correction to protect against Type I errors. The OSCE score was regarded as a quasi-continuous variable to perform statistical analysis. Analyses were performed using *JMP* (version 10.0.2, 64 bit, SAS Institute Inc., NC, USA). ICC(3,1) was calculated using *R* (Version 3.0.0) and the *irr* package *(*extension package for the R software, in order to calculate the inter-class correlation) [22, 23]. Graphics were created using *Prism* (Version 6.01, GraphPad Software, Inc., CA, USA).

### Ethical considerations

Participation was voluntary, and written informed consent was obtained from all participants. All participants in this study received a small gift after completion. Students not participating in this study had no disadvantages regarding the BLS course. The study was approved by the ethical committee of the University of Tübingen (Reference 539/2012) and conducted in accordance to the Declaration of Helsinki (Seoul 2008).

## Results

### Baseline characteristics

Inter-rater agreement was high with a median ICC of .83 for the checklist items individually assessed by two raters. The characteristics of the students are shown in Table 1. Five datasets were excluded because of technical problems with the manikin or video recording (three in the X3, one in X5 and X8). In a post-hoc power analysis, we could detect a 4-point OSCE score difference with n = 18 for each group, α = .05, 1 – β = .8 and standard deviation = 2.84.

**Table 1:** **Baseline characteristics and demographics**

	All students	X3	X5	X8
Total (n)	69	27	19	23
Age, Median (IQR)	24 (23 – 28)	24 (23 – 27)	23 (22 – 27)	24 (23 – 28)
Female, n (%)	41 (59%)	16 (59%)	14 (73%)	11 (47%)
Months since last resuscitation training, median (IQR)	33 (23 – 45)	37 (24 – 47)	36 (22 – 45)	32 (22 – 43)
Paramedical qualification, n (%)	10 (14%)	3 (11%)	3 (16%)	4 (17%)
Other medical qualification, n (%)	11 (16%)	4 (15%)	2 (11%)	5 (7%)

Baseline characteristics and demographics for all students included in the data analysis. Values are shown for all students and for students in groups of three (X3), five (X5) or eight students per tutor (X8).

### OSCE assessments and resuscitation quality

**Figure 2:**
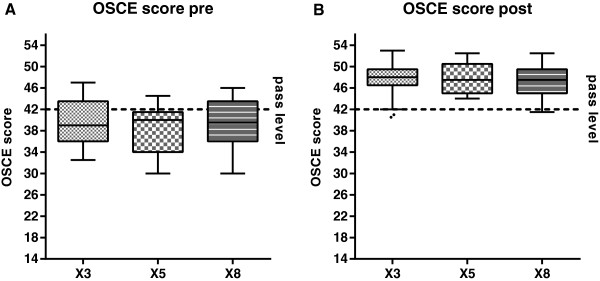
**OSCE scores pre- and post-test.** OSCE scores at pre- **(A)** and post-test **(B)** in groups of three (X3), five (X5) or eight students per tutor (X8). Before the BLS training, many students were below the pass rate of 42 points in total (dashed line). After the training, the vast majority of students from all groups passed the OSCE.

In the baseline assessment, we found comparable OSCE scores in X3, X5 and X8 (Figure 2, X3: 39.0 (IQR 36.0 – 43.5), X5: 40.0 (IQR 34.0 – 41.5), X8: 39.5 (IQR 36.0 – 43.5), p = .46), corresponding to pass rates of 41%, 16% and 35% at a pass level of 42 points). OSCE scores at post-test were similarly high among groups of three, five or eight students per tutor (X3: 48.0 (IQR 46.5 – 49.5), X5: 47.5 (IQR 45.0 – 50.5), X8: 47.5 (IQR 45.0 – 49.5), p = .96), corresponding to pass rates of 93%, 100% and 100%. Chest compression rates and depths at post-test were mostly within the recommended ranges. We detected no significant differences in the number of students that were within the recommended ranges for compression depth and frequency. All objective resuscitation parameters are reported in Table 2.

**Table 2:** **Objective resuscitation quality**

Variable	Assessment	All students	X3	X5	X8
Compression depth [mm] (mean ± SD)	Pre	48.2 ± 10.7	50.1 ± 10.0	40.5 ± 11.1	52.4 ± 7.7
	Post	54.5 ± 5.3	55.6 ± 6.1	52.7 ± 4.7	54.8 ± 4.5
Compression depth (% within range)	Pre	31,9%	40,7%	15,8%	34,8%
	Post	69,6%	55,6%	78,9%	78,3%
Compression rate [1/min] (mean ± SD)	Pre	102.8 ± 21.9	106.0 ± 22.6	98.6 ± 21.6	102.2 ± 21.7
	Post	113.6 ± 11.1	112.9 ± 10.9	108.6 ± 8.0	118.5 ± 11.9
Compression rate (% within range)	Pre	29,0%	33,3%	21,1%	30,4%
	Post	66,7%	70,4%	78,9%	52,2%
Duty Cycle [%] (mean ± SD)	Pre	46.5 ± 7.5	45.1 ± 8.0	49.5 ± 7.2	45.6 ± 6.6
	Post	45.4 ± 5.0	45.0 ± 6.1	45.3 ± 4.6	46.0 ± 3.8
Compressions completely released [%] (median (IQR))	Pre	99 (73 – 100)	99 (67 – 100)	99 (47 – 100)	98 (86 – 100)
	Post	96 (65 – 99)	97 (60 – 100)	95 (69 – 99)	97 (59 – 99)

Objective resuscitation quality, reported as proposed by Kramer-Johansen *et al.* [20]. Values are shown for all students and for students in groups of three (X3), five (X5) or eight students per tutor (X8). For compression depth and rate, we furthermore report the % of students within the recommended range [1].

### BLS teaching session

**Figure 3:**
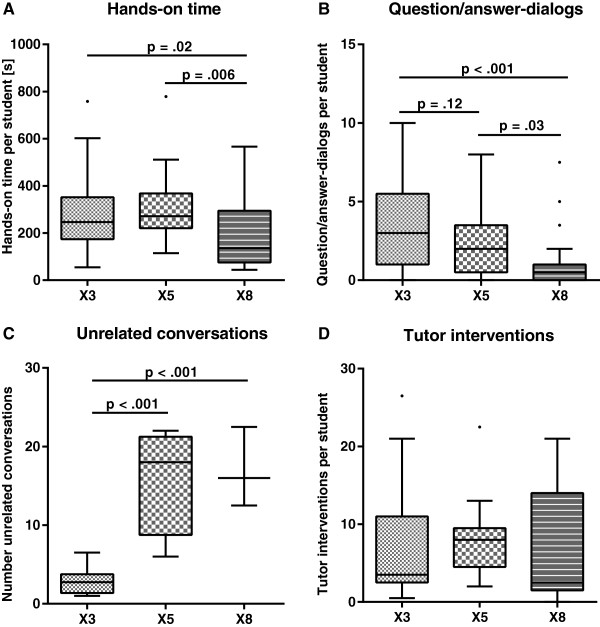
**Effect of group size on hands-on time, questions, unrelated conversation and tutor interventions.** Graphical representation of teaching observations during the BLS training session for groups of three (X3), five (X5) and eight students per tutor (X8). Tukey box-and-whisker plots contain the median and represent the lower and upper quartile. **A**. Students in groups of eight participants per tutor had less hands-on time. **B**. The amount of question/answer-dialogs decreased in larger groups. **C**. Groups with five and eight students each conducted more unrelated conversations than groups of three students per tutor. **D**. The teaching interventions by a tutor (e.g. correcting a wrong hand placement during chest compression) did not differ between the different groups.

We found statistically significant differences in the time for each individual participant to practice BLS skills. The effective individual hands-on time was comparable for participants in groups of three and five students, but lower for participants in groups of eight students per tutor (X3: 4:07 min (IQR 2:54 - 5:52 min), X5: 4:32 min (IQR 3:41 - 6:08 min), X8: 2:16 min (IQR 1:15 - 4:55 min, X3 versus X8: p = .02, X5 versus X8: p = .006, Figure 3A). Participants in groups of eight students per tutor were less likely to ask questions than classmates in groups of three students per tutor (number of question/answer-dialogs per student in X3: 3.0 (IQR 1.0 - 5.5), X5: 2.0 (IQR 0.5 - 3.5), X8: 0.5 (IQR 0.0 - 1.0), X3 versus X8: p < .001, Figure 3B). Groups of five or eight students were more likely to conduct unrelated conversations than groups of three students (number of unrelated conversations in group X3: 2.9 ± 1.7, X5: 16.0 ± 7.0, X8: 17.0 ± 5.1,, X3 versus X5: p < .001, X3 versus X8: p < .001, Figure 3C). The teaching interventions by the tutor for each student were comparable among the groups (X3: 3.5 (IQR 2.5 - 11.0), X5: 8.0 (IQR 4.5 - 9.5), X8: 2.5 (IQR 1.5 - 14.0), Figure 3D).

### Self-assessment

Before the training, participants self-assessments were comparable among the groups (X3: 3.0 (IQR 2.0 - 4.0), X5: 3.0 (IQR 2.0 - 5.0), X8: 3.0 (IQR 2.0 - 6.0), p = .82). Following the training session, students in groups of eight classmates had a lower self-assessment than participants in groups of three or five classmates (X3: 8.2 (IQR 7.2 - 9.0), X5: 8.0 (IQR 7.9 - 9.0), X8: 7.0 (IQR 6.1 - 9.0). X3 versus X8: p = .007, X5 versus X8: p = .03).

## Discussion

In this prospective, randomized and double-blind simulation study, we investigated the impact of different group size on the effectiveness of resuscitation training and on the training session itself. We found that teaching BLS skills was effective in groups of three, five or eight medical students per tutor. After the training, all groups had high OSCE pass rates of over 90% and had comparable OSCE scores. However, we observed a reduced individual teaching focus in larger groups. Although tutors were told to spend the same time practicing for each individual participant, students in groups of eight classmates had less hands-on time to practice BLS skills. This difference might be due to a higher proportion of “observational learning” or due to a subjective time pressure in the larger groups. Furthermore, students in larger groups were less likely to ask questions and had a lower subjective self-assessment.

The results of this study have important practical implications. For teachers at medical schools or in paramedic education, assigning a certain number of students to a tutor is an everyday challenge. In doing so, it is important to find a balance between a teacher-intensive small group setting and classes with more students per tutor. Although evidence to support this decision is limited, this work provides an additional reference for teachers to make an evidence-based choice. While we could not detect a difference in gain of performance in larger groups of eight students, there was a decline in teaching quality (less hands-on time and more unrelated conversation). Our study population already had fairly good resuscitation skills in the pre-test, so there might be a ceiling effect. Thus it is justifiable to teach BLS skills with medical students in group sizes up to eight students per tutor if smaller groups are not feasible. For more complex skills or a less advanced audience, the decline in teaching quality in larger groups may lead to a suboptimal teaching result.

In our study, the majority of all participants did not pass our OSCE before receiving the standardized BLS training. In the post-test assessment, more than 90% of students passed the OSCE. Although this effect can be explained partially by the fact that the students performing the post-test OSCE already knew the scenario and had some activation of prior knowledge from the pre-test, this effect alone is unlikely to be sufficient to explain that most students pass the second OSCE. Aside from the fact that the resuscitation quality was already included in the OSCE checklist, we also assessed the quality parameters individually. The objective resuscitation quality increased in all groups from the pre- to the post-test, still we observed variances in the different group sizes. The main learning success is indicated by the checklist items assessing clinically important aspects as the general pace, the assessments and the ventilation success [1]. Another important finding was that pass rates in the post-test were comparable among groups of three, five or eight students per tutor. This has important medical implications, because one could state the students from all groups are “safe to practice” [24].

There is evidence from interactive education sessions that the student participation is lower in larger groups [15]. Looking at the teaching session itself, we found that the amount of questions asked by students decreased as group size increased. This could be explained partially by the fact that some questions are also asked by other classmates in larger groups. This effect might account for some questions, but not all questions a student intends to ask. To confirm this, we calculated the amount of questions asked per minute and found that it was highest in groups of three and lowest in groups of eight students per tutor. Consequently, the amount of unrelated conversations conducted within the groups increased with group size. This finding further supports the results of Wheelan *et al.* who reported that students in larger groups participate and concentrate less than students in smaller groups [15]. However, we only counted the number of topic related/unrelated conversations and did not perform an in-depth analysis whether the conversation had an additional positive or negative effect (i.e. improvement of group dynamics).

This lower participation might have some implications on the students’ contentment and self-assessment. Kooloos *et al.* found a lower student satisfaction in larger groups compared with smaller groups [25]. The lower contentment could lead to a negative *Hawthorne effect*, where students in larger groups feel worse and therefore perform worse [26]. Although we did not measure the satisfaction of our participants, we found that students in groups of eight classmates had a lower self-assessment than participants in groups of three or five students per tutor. Remarkably, this is despite the fact that all groups had a comparable OSCE score. Therefore, the increased group size already had an influence on the perceived learning success but not yet on the performance of the medical students.

To our knowledge, this is the first prospective double blind study to investigate the impact of group size on the effectiveness of a resuscitation curriculum measuring the resuscitation outcome with an OSCE. Our results are in agreement with previous data, although Rezmer *et al.* only investigated students in relatively small groups of two, three or four students per tutor in a retrospective survey and post-simulation exam [14]. In contrast, Cooper *et al.* focused on less practical and more theoretical skills like taking a patient’s history [27]. In their investigation, smaller groups seemed to perform better than larger groups [27]. While our results are true for skills such as BLS, they may not be applicable for more complex skills as required for advanced life support resuscitations. In more sophisticated settings, or in other target groups as medical students, smaller groups might benefit from the ability to ask questions and to focus on the teaching session.

Strengths of this study are the prospective, randomized and double-blind approach as well as the acquisition of distinct outcomes: a checklist, objective manikin data (as checklists are not sensitive for identifying poor chest compression quality [28]) and observations recorded during the teaching session itself. We used video- and audiotaping to conduct the study in a double-blinded fashion. Although the awareness of being video recorded might change the behavior of the participants and tutors, there is evidence that many people quickly forget that they are being recorded [29]. A sophisticated resuscitation manikin provided objective chest compression performance. Although we cannot provide ventilation performance due to inaccurate manikin measurements in the breathing parameters, the total ventilations count and the average ventilation success as observed by the video raters are included in the OSCE score. Another shortcoming of our investigation is that a total of 36 students declined to participate, which could result in a possible selection bias. Though OSCE scores were comparable among the different group sizes, there were some variations in objective chest compression parameters. More focus in general should be put on objective resuscitation quality because it is known to be poor even for professional helpers [3]. As we only evaluated medical students, it needs to be investigated if our results can be transferred to other groups like lay-rescuers or paramedic staff.

## Conclusions

This research showed that group size up to eight students did not influence the teaching success of BLS skills when measured on a distinct checklist to assess resuscitation skills. However, smaller groups yielded a longer practicing time, had more opportunities to ask questions and conducted less unrelated conversations. Therefore these results warrant careful interpretation, as smaller groups may be advantageous for more complex skills. Further studies are necessary to assess the impact of group size on the teaching of more complex skills and on long-term retention of the skills or teaching a less advanced audience.

## Electronic supplementary material

Additional file 1: **Teaching plan.** The teaching plan that was used during the BLS sessions. The plan was originally used in German language and was translated for publication. (DOCX 152 KB)

Additional file 2: **OSCE checklist and guide for marking.** The checklist used for the OSCE rating as well as the guide for marking, containing information about how each item should be rated. (PDF 493 KB)
