# Supplementary material for: Basic life support is effectively taught in groups of three, five and eight medical students: a prospective, randomized study
Source: BMC Med Educ. 2014 Sep 6;14:185. doi: 10.1186/1472-6920-14-185 (PMC4168208; doi:10.1186/1472-6920-14-185)
Supplement: Supplementary file 1 — Additional file 1: Teaching plan. The teaching plan that was used during the BLS sessions. The plan was originally used in German language and was translated for publication. (DOCX 152 KB) [file 12909_2013_1015_MOESM1_ESM.docx]

Teaching plan Basic Life Support

# Theory (5 min)

- Introduction
- Causes of cardiac arrest (Heart failure, embolism, etc.)
- Implications for therapy (early therapy, brain death, etc.).
- Resuscitation algorithm

# Hands on: Assessment (5 min)

Explain theory. Then, demonstrate at least 2 times

1. Self-protection (Wear gloves)
2. Assess consciousness (shake, check response)
3. Open airway (Chin lift, head tilt)
4. Check breathing (see, listen, feel)
5. Emergency call

# Hands on: Chest compression (5 min)

Explain theory. Then, demonstrate at least 2 times

1. Knee next to patient
2. Hands on the middle of the chest
3. Elbows straight
4. Have your body above the chest
5. Chest compression, 5 - 6cm
6. Complete release
7. Repeat 30x with frequency 100/min – 120/min

# Hands on: Ventilation (5 min)

Show and explain ventilation bag. Then, demonstrate at least 2 times

1. After 30x chest compressions: Open airway (Head tilt, chin lift)
2. Knee behind patients‘ head
3. Fit mask
4. Ventilate
   1. Chest raise in 1 sec
   2. Passive chest lowering in 1 sec
5. Repeat
6. Do not spend more than 5 secs on ventilations
7. Continue CPR directly afterwards

# Demonstrate algorithm (3 min)

Explain algorithm again, then demonstrate.

1. Assessment
2. Demonstrate 5 times of 30:2

# Training (ca. 6 min per student)

**Stick to order of the students.**

**While taining, validate chest compression using manikin software.**

1. Assessment isolated
2. Chest compressions isolated
3. Ventilation isolated
4. Then, whole algorithm: Assessment and 5 times 30:2
