# Supplementary material for: Basic life support is effectively taught in groups of three, five and eight medical students: a prospective, randomized study
Source: BMC Med Educ. 2014 Sep 6;14:185. doi: 10.1186/1472-6920-14-185 (PMC4168208; doi:10.1186/1472-6920-14-185)
Supplement: Supplementary file 2 — Additional file 2: OSCE checklist and guide for marking. The checklist used for the OSCE rating as well as the guide for marking, containing information about how each item should be rated. (PDF 493 KB) [file 12909_2013_1015_MOESM2_ESM.pdf]

PocLab Simulationszentrum
Medizinische Fakultät Tübingen

EBERHARD KARLS UNIVERSITÄT TÜBINGEN

BLS-Checklist

1 Safe approach
4: Gloves from beginning
3: Gloves before CPR was started
2: Gloves later
1: No gloves worn

2 Check for responsibility
4: Shaking **and** Talking
3: Shaking **or** Talking
2: Other approach
1: No responsibility checked

3 Open airway
4: Head tilt, chin lift
3: Head tilt OR chin lift
2: Airway open, but other attempt
1: Airway not opened

4 Check for breathing
4: Look, listen and feel
3: Only two elements (e.g. look and feel)
2: Only one element or other element
1: No check for breathing

5 Call for help using mobile
4: Call for help directly after no breathing is detected
3: Call for help before CPR is started
2: Call for help later
1: No call for help

6 CPR is started
4: Yes, immediately after last action (< 5 sec)
3: Yes, very fast after last action (< 10 sec)
2: Yes, prolonged
1: No

7 Ventilation/compression ratio
4: 30:2 always
3: 27:2 – 33:2 always
2: Compressions only
1: Other

8 Hand-placement during compression
4: Center of chest always
3: Center of chest most of the time
2: Center of chest sometimes
1: Wrong

9 Average compression depth*
4: 50 – 60 mm
3: 45 – 49 mm OR 61 - 65 mm
2: 40 – 44 mm OR 66 - 71 mm
1: Other

10 Average compression rate*
4: 100/min – 120/min
3: 90 – 99/min OR 121 – 130/min
2: 80 – 89/min OR 131 – 140/min
1: Other

11 Percentage completely released compressions*
4: 90% and above
3: 80% and above
2: 70% and above
1: Below

12 Total ventilations counted
4: 10
3: 9 - 11
2: 8 – 12
1: Other

13 Average ventilation success
4: Chest rises always
3: Chest rises most of the time
2: Chest rises sometimes
1: No chest rise

14 Compression pause for ventilations
4: < 5 sec always
3: < 5 sec most of the time
2: < 10 sec most of the time
1: Other

*** Values obtained from LAERDAL software.**

Always: At least 90% of the time.
Most of the time: At least 80% of the time.

Guide for marking

1 Safe approach

2 Check for responsibility

3 Open airway
Check if head tilt and chin lift are correctly performed by the participant.

Image placeholder (not shown due to copyright restrictions).

4 Check for breathing
4: Look, listen and feel
3: Only two elements (e.g. look and feel)
2: Only one element or other element
1: No check for breathing

5 Call for help using mobile
The trainee has to announce an emergency call. It is **not** necessary to simulate a talk.

6 BLS is started

7 Ventilation/compression ratio

8 Hand-placement during compression
The hand must be placed in the center of the chest = lower half of the sternum.

Image placeholder (not shown due to copyright restrictions).

9 Average compression depth*
This parameter is digitally calculated.

10 Average compression rate*
This parameter is digitally calculated.

11 Percentage completely released compressions*
This parameter is digitally calculated.

12 Total ventilations counted
Please count the total ventilations attempts. It is not necessary that the ventilations have an effect (see below).

13 Average ventilation success
For a successful ventilation, the chest of the CPR manikin has to rise.

14 Average compression pause for ventilations
Please stop the pause between the last compression before and the first compression after ventilations.

Training

Training time (min)
The training time starts as soon as the trainee sits right next to the manikin. It is interrupted only if the trainee loses focus of the tutor (e.g. if the tutor interacts with other trainees than the actual one). The training time ends as soon as the trainee leaves the manikin.

Tutor-Interventions
A tutor intervention is a verbal, visual or manual hint by the tutor that is intended to increase the trainee's abilities. Example: Tutor says "Push a bit deeper"; Tutor shows how to use the ventilation bag; etc. If the tutor combines two "hints" in one sentence, this counts as two Tutor-interventions. Every hint by the tutor that implies a behavioral change of the trainee is counted as a intervention.

Question/Answer-Dialog
A question/answer-Dialog is a dialog between tutor and trainee. It consists of a question from the trainee to the tutor and it's matching answer by the tutor. Every question is counted.
